# Electrical and Structural Properties of Li_1.3_Al_0.3_Ti_1.7_(PO_4_)_3_—Based Ceramics Prepared with the Addition of Li_4_SiO_4_

**DOI:** 10.3390/ma14195729

**Published:** 2021-09-30

**Authors:** Konrad Kwatek, Wioleta Ślubowska, Jan Leszek Nowiński, Agnieszka Teresa Krawczyńska, Isabel Sobrados, Jesús Sanz

**Affiliations:** 1 Faculty of Physics, Warsaw University of Technology, 00-662 Warsaw, Poland; wioleta.slubowska@pw.edu.pl (W.Ś.); jan.nowinski@pw.edu.pl (J.L.N.); 2 Faculty of Materials Science and Engineering, Warsaw University of Technology, 02-507 Warsaw, Poland; agnieszka.krawczynska@pw.edu.pl; 3 National Research Council, The Materials Science Institute of Madrid (ICMM CSIC), 28049 Madrid, Spain; isobrado@icmm.csic.es (I.S.); jsanz@icmm.csic.es (J.S.)

**Keywords:** ceramic, composite, NASICON, Li_1.3_Al_0.3_Ti_1.7_(PO_4_)_3_, ionic conductivity, sintering agent

## Abstract

The currently studied materials considered as potential candidates to be solid electrolytes for Li-ion batteries usually suffer from low total ionic conductivity. One of them, the NASICON-type ceramic of the chemical formula Li_1.3_Al_0.3_Ti_1.7_(PO_4_)_3_, seems to be an appropriate material for the modification of its electrical properties due to its high bulk ionic conductivity of the order of 10^−3^ S∙cm^−1^. For this purpose, we propose an approach concerning modifying the grain boundary composition towards the higher conducting one. To achieve this goal, Li_4_SiO_4_ was selected and added to the LATP base matrix to support Li^+^ diffusion between the grains. The properties of the Li_1.3_Al_0.3_Ti_1.7_(PO_4_)_3_−*x*Li_4_SiO_4_ (0.02 ≤ *x* ≤ 0.1) system were studied by means of high-temperature X-ray diffractometry (HTXRD); ^6^Li, ^27^Al, ^29^Si, and ^31^P magic angle spinning nuclear magnetic resonance spectroscopy (MAS NMR); thermogravimetry (TG); scanning electron microscopy (SEM); and impedance spectroscopy (IS) techniques. Referring to the experimental results, the Li_4_SiO_4_ additive material leads to the improvement of the electrical properties and the value of the total ionic conductivity exceeds 10^−4^ S∙cm^−1^ in most studied cases. The factors affecting the enhancement of the total ionic conductivity are discussed. The highest value of *σ_tot_* = 1.4 × 10^−4^ S∙cm^−1^ has been obtained for LATP–0.1LSO material sintered at 1000 °C for 6 h.

## 1. Introduction

In the next generation of lithium-ion batteries, the use of organic liquid electrolytes should be avoided because of safety issues. These electrolytes have low thermal stability and high flammability. They can cause fire accidents as well as explosions if batteries are improperly used or stored. Therefore, all-solid-state batteries (ASSBs) are considered as a good choice to replace conventional Li-ion batteries. In ASSBs, the liquid electrolyte is replaced by a solid one not only to address safety issues (solid electrolytes are thermally more stable) but also because it has other advantages over liquid electrolytes [1,2,3,4].

First of all, lithium-ion batteries with solid electrolytes have better mechanical properties. Secondly, they have a wider electrochemical window that makes them compatible with a greater number of possible cathode materials, especially with those of higher potential vs. lithium metal (>4 V), which increases energy density. Lastly, with solid electrolytes, the Li-ion battery packaging can be simplified and dead weight can be considerably reduced, resulting in increased energy density. However, the material used as a solid electrolyte should meet several requirements. It should possess high ionic conductivity above 10^−4^ S/cm at room temperature; should have negligible electronic conductivity with a high ionic transference number; and should have a wide electrochemical stability window. Several types of Li-ion solid electrolytes can satisfy the above-mentioned requirements, including the NASICON-type, garnet-type, perovskite-type, LISICON, LiPON, Li_3_N, sulfide, argyrodite, anti-perovskite, etc. [1,2,3,4].

Among all NASICON-type Li-ion conductors, LiTi_2_(PO_4_)_3_ (LTP) has the most suitable skeleton for Li-ion diffusion. It crystallizes in a NASICON-type structure with rhombohedral symmetry and belongs to the R-3c space group. In the NASICON-type structure, TiO_6_ octahedra and PO_4_ tetrahedra are linked by their corners to form a 3D stable network [5,6,7,8]. However, its total ionic conductivity is still low because of highly resistive grain boundaries and low sinterability [8,9,10,11,12,13,14,15]. To address that issues, the partial replacement of Ti^4+^ ions by trivalent Al^3+^ ions was adopted as a way to improve sinterability and enhance the ionic conductivity. Li_1.3_Al_0.3_Ti_1.7_(PO_4_)_3_ (LATP) displayed a total ionic conductivity as high as 10^−4^ S/cm at room temperature, depending on the preparation procedure [8,9,10,11,12,13,14,16,17,18,19,20,21,22]. Moreover, the compound exhibits good thermal and mechanical stability, good chemical stability against moisture in air, negligible electronic conductivity, and low costs, making it suitable for large-scale production. 

Grain boundary conductivity of LATP was found to be highly dependent on the sintering process, resulting in different particle size distributions (i.e., microstructure) and different impurity phases [8,10,11,14,23,24,25,26]. The effectiveness of the LATP sintering process can be considerably improved by the use of appropriate sintering agents, such as LiF [14,27,28], Li_3_PO_4_ [29], Li_3_BO_3_ [24,29], LiBO_2_ [30], LiBF_4_ [31], LiNO_3_ [32], Li_2_O [33], and Li_4_SiO_4_ [this work]. These inorganic salts are known to affect the microstructure (lower porosity) and secondary phase formation, leading to higher total ionic conductivity. In order to increase the total conductivity, the grain-boundary resistivity should be lowered. One possible approach is to decrease the thickness of grain boundaries by promoting the growth of large grains [11,20,34,35]. However, the increase of grain size alone is not sufficient. This is why the other approach is to modify the grain-boundary composition towards a higher conducting one [23,24,29,30,33].

In this work, we adopt the latter approach in order to obtain high conductive materials. For this purpose, we introduce the Li_4_SiO_4_ additive into the LATP matrix. The choice of lithium orthosilicate was dictated by the fact that it is a lithium-ion conductor and its presence at grain boundaries supports Li^+^ diffusion between LATP grains. Thus, we expect that the value of the total ionic conductivity should exceed 10^−4^ S/cm and be higher than LATP ceramics [23,24], while maintaining thermal and chemical stability.

## 2. Materials and Methods

Polycrystalline Li_1.3_Al_0.3_Ti_1.7_(PO_4_)_3_ and Li_4_SiO_4_ compounds were obtained via a conventional solid-state reaction method. Reagent-grade chemicals, Li_2_CO_3_ (Sigma Aldrich, Saint Louis, MO, USA), NH_4_H_2_PO_4_ (POCh), anatase TiO_2_ (Sigma Aldrich), Al_2_O_3_ (Sigma Aldrich), and SiO_2_ (Sigma Aldrich) were weighted in stoichiometric amounts and then ground with a mortar and pestle. The Li_1.3_Al_0.3_Ti_1.7_(PO_4_)_3_ material was synthesized in an alumina crucible at 900 °C for 10 h, while the Li_4_SiO_4_ material was synthesized at 900 °C for 8 h. Subsequently, the obtained polycrystalline-milled Li_4_SiO_4_ powder was added to as-prepared LATP material in a molar ratio varying from 2 to 10%. Next, both components were ball-milled in ethanol at 400 rpm for 1 h, dried, and pelletized under uniaxial 10 MPa pressure. Finally, pellets 6 mm in diameter and ca. 2 mm thick were formed and sintered at 800, 900, or 1000 °C for 2, 6, or 12 h.

The phase composition of the as-prepared materials and composite powders after heat treatment were examined by means of the X-ray diffraction method. Data were collected in the *2θ* range from 10° to 90° with a 0.033° step size and a counting rate of 100 s per step with CuKα line using a Philips X’Pert Pro diffractometer. Additionally, temperature-dependent XRD (HTXRD) patterns were recorded in the temperature range of 30–800 °C using an Anton Paar HTK−1200 oven. 

The thermal stability of the composites was determined by thermal gravimetric analysis (TGA). A TA Instruments Q600 calorimeter was used to register the mass loss as a function of temperature (with reference to an empty alumina crucible) during heating under airflow in the temperature range of 50–1000 °C. The measurements were performed at the heating rate of 10 °C·min^−1^ on ca. 20 mg powdered samples. 

The cross sections of the freshly fractured pellets were polished by the ion milling system IM 4000 and subsequently were observed using the scanning electron microscope (SEM) SU 8000 Hitachi at 5 kV in secondary electron (SE) and backscattered electron (BSE) modes. 

For impedance spectroscopy measurements, both bases of the as-formed pellets were polished with sandpaper and covered with Pt as electrodes. Impedance investigations were carried out using a Solartron 1260 frequency analyzer in the frequency range of 1–10^7^ Hz and in the temperature range of 30–100 °C during both heating and cooling runs. The impedance data was collected using a self-developed software [36].

^27^Al, ^6^Li, ^29^Si, and ^31^P MAS NMR spectra were recorded with a Bruker AVANCE-400 spectrometer (9.4T magnetic field) and the resonance frequencies were 104.3, 58.9, 79.5, and 162.0 MHz, respectively. For single-pulse ^27^Al and ^6^Li MAS NMR spectra, π/6 pulses of 2 μs were used, but for ^31^P and ^29^Si spectra, 4 μs irradiation (π/2 pulses) were applied. The choice of π/6 pulses for ^27^Al MAS NMR signals was adopted for the quantification of Al components [37,38]. The MAS technique (rotation of samples at 10 kHz around an axis inclined at 54°44′ with respect to the external magnetic field) was used with a Bruker MAS NMR probe with a 4 mm (outer diameter) ZrO_2_ rotor at a spinning frequency of 10 kHz. A recycle delay of 5 s was chosen in the case of ^27^Al and ^6^Li, while 20 s and 60 s were used in the case of ^29^Si and ^31^P, respectively. The spectra were collected with 24, 80, 800, and 12,000 scans for ^31^P, ^27^Al, ^6^Li, and ^29^Si, respectively. Chemical shift values of NMR resonances were referred to 1 mol·L^−1^ AlCl_3_, 1 mol·L^−1^ LiCl, TMS, and 85 wt.% H_3_PO_4_ aqueous solutions. The NMR spectra were simulated using the *dmfit* software [39]. The accuracy of the chemical shift was ±0.1 ppm.

## 3. Results

### 3.1. X-ray Diffraction

**Figure 1:**
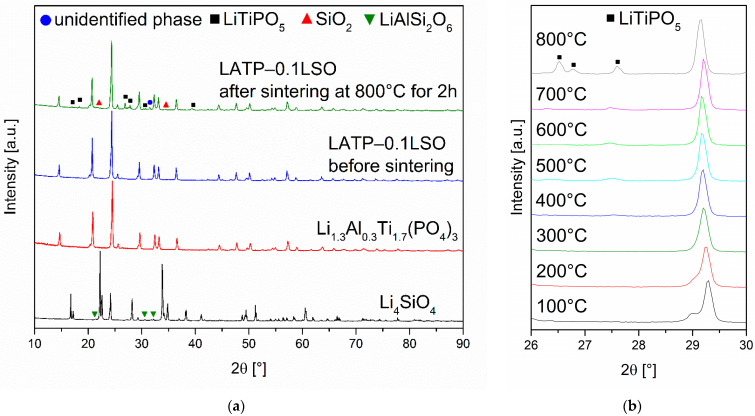
(**a**) XRD patterns of the as-prepared Li_1.3_Al_0.3_Ti_1.7_(PO_4_)_3_ and Li_4_SiO_4_ materials, and the LATP–0.1LSO composite before and after sintering at 800 °C. (**b**) HTXRD patterns of the LATP–0.1LSO composite collected in the temperature range of 100–800 °C with 100 °C step.

X-ray diffraction patterns of the LATP-0.1LSO composite before and after sintering are given in Figure 1a. For comparison, X-ray diffraction patterns of the synthesized Li_1.3_Al_0.3_Ti_1.7_(PO_4_)_3_ and Li_4_SiO_4_ materials are also included. In the latter case, besides the reflections assigned to Li_4_SiO_4_, some additional weak lines are also present, corresponding to LiAlSi_2_O_6_. When both of the components were mixed, no significant differences were observed between the diffraction patterns of the LATP and LATP-0.1LSO materials. However, when the composite material underwent the heat treatment process, some additional diffraction peaks emerged at *2θ* angles: 17.1°, 18.3°, 27.0°, 27.7°, 30.6°, and 39.6°. They were assigned to the LiTiPO_5_ phase [23,24,40,41,42]. Apart from the peaks of these phases, other diffraction peaks at *2θ* angles around 21.8° and 31.6° were detected. The former may be attributed to the SiO_2_ phase, while the weak line at *2θ* angles around 31.6° may be assigned to some unidentified phase.

In order to perform a more detailed study on the phase composition, high-temperature X-ray diffractometry was used (Figure 1b). The analysis of the collected data reveals that at about 500 °C, one additional peak at *2θ* angles ca. 27.5° started to emerge. This may be associated with an intermediate product of the LiTiPO_5_ compound, which was completely formed around 800 °C [23,24,41]. No additional phases were detected during the HTXRD investigations.

### 3.2. Thermal Analysis

**Figure 2:**
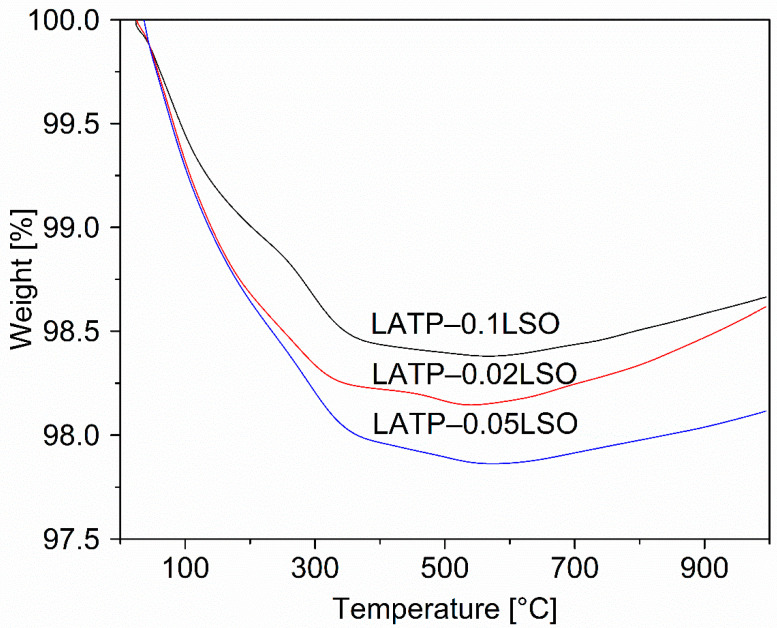
TG plots
recorded on LATP–LSO composites.

The thermal stability of the as-prepared non-sintered components and composites were studied using thermogravimetry. Figure 2 illustrates the weight loss vs. temperature for the studied composites. One can observe that the highest mass loss (ca. 2%) was produced at the beginning of the heating ramp, up to around 300 °C. Such mass loss may be assigned to the evaporation of the water moisture and residual ethanol (used during milling of the components) retained at the grain boundaries. However, at ca. 530 °C, the samples’ weight slightly increased (ca. 0.3%), which may have resulted from the formation of secondary phases, namely for LiTiPO_5_.

### 3.3. Microstructure

**Figure 3:**
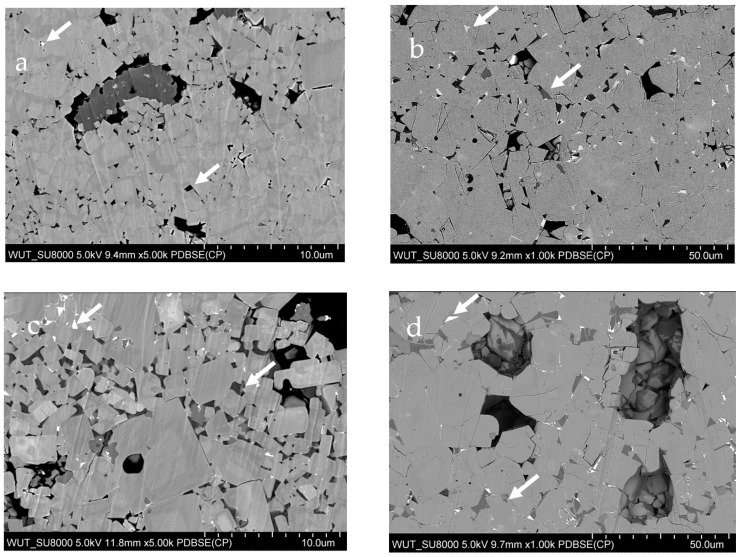
SEM images of the LATP–0.02LSO sintered at 800 °C for 2 h (**a**) and 1000 °C for 12 h (**b**), and LATP–0.1LSO sintered at 800 °C for 2 h (**c**) and 1000 °C for 12 h (**d**). Brighter/darker regions marked with white arrows concern secondary phases (SiO_2_ and LiTiPO_5_).

The SEM images taken in the BSE mode of LATP–0.02LSO sintered at 800 °C for 2 h and 1000 °C for 12 h, and LATP–0.1LSO sintered at 800 °C for 2 h and 1000 °C for 12 h are presented in Figure 3. Microstructures observed in the SE mode are presented in Figure S1. One may notice that changes in the microstructure are correlated with sintering temperature. The microstructure of the LATP–0.02LSO material sintered at 800 °C (Figure 3a) was composed of ca. 1–2 μm, irregular in shape and size grains. There are also a few pores and the neighboring grains did not adhere to each other very well. One may notice secondary phases (marked as brighter/darker regions), which may be related to the SiO_2_ and LiTiPO_5_ compounds. When sintering temperature was increased to 1000 °C (Figure 3b), the grains became bigger and more densely packed. However, one may still observe smaller (ca. 10 μm) and bigger (ca. 20 μm) grain sizes. The number of pores was lower and they became smaller than in the previous case. The analysis of the images of LATP–0.1LSO samples (Figure 3c,d) leads to a similar conclusion, which concerns the formation of dense ceramic when the composite undergoes heat treatment at higher temperatures. While the material undergoes a heat treatment at 800 °C, the area of brighter/darker regions increases with the concentration of the LSO. However, when the material is sintered at 1000 °C, regardless of the content of the additive, no significant changes in the concentration of the regions related to secondary phases are observed. Also, it is worthy to mention that for composites sintered at 1000 °C, the microstructure consisted of microcracks through LATP grains, which may have occurred due to rapid grain growth.

### 3.4. MAS NMR

The ^6^Li, ^27^Al, ^29^Si, and ^31^P MAS NMR studies were performed on LATP–0.1LSO composites (sintered at 900 and 1000 °C), providing accurate information about the compositional and structural changes taking place during sintering.

**Figure 4:**
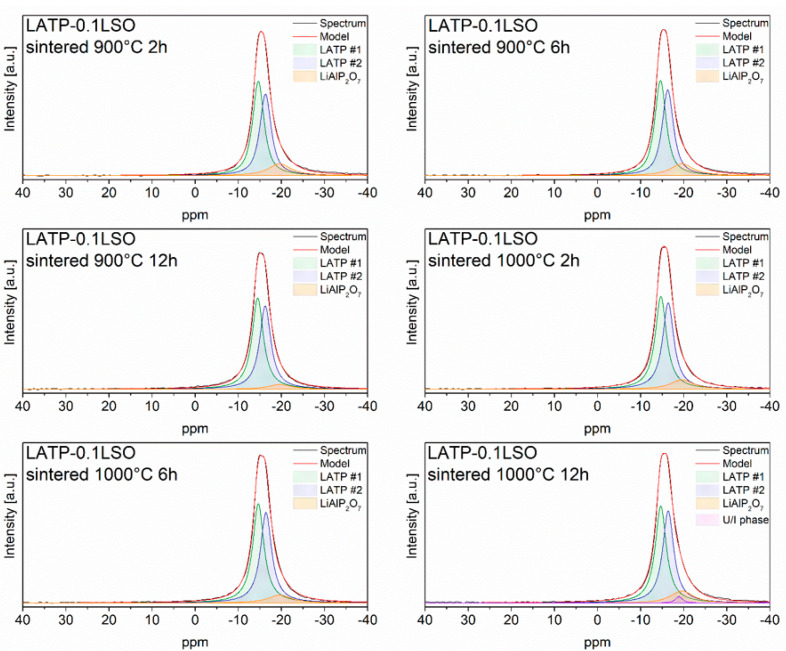
^27^Al MAS NMR spectra of LATP–0.1LSO sintered at 900 and 1000 °C for 2, 6, or 12 h. The experimental and simulated spectra are displayed as black and red lines, respectively. The simulated spectra are the sum of distinct line-shapes displayed as green, purple, and orange lines.

^27^Al MAS NMR spectra (Figure 4) of the LATP–0.1LSO material was composed of one asymmetric and broad resonance at about −15 ppm. This band could be deconvoluted into at least three signals at −14.6, −16.3, and −19.6 ppm, corresponding to the three octahedral (AlO_6_) environments [5,23,41,42,43,44,45,46]. The two closely located signals at −14.6 and −16.3 can be assigned to the Li_1+x_Al_x_Ti_2−x_(PO_4_)_3_ phase. In such a case, their occurrence may be related to the existence of two NASICON-type phases with slightly different chemical compositions [24,42,47,48]. The last resonance at −19.6 ppm may have been due to the presence of a LiAlP_2_O_7_ compound [24,41,42]. For the LATP–0.1LSO material sintered at 1000 °C for 12 h, one additional ^27^Al NMR signal at −18.9 ppm may be found and ascribed to some unidentified phase containing aluminum in octahedral coordination. Referring to different sintering conditions, one can notice only slight changes in the ^27^Al MAS NMR spectra. Considering the relative integrated intensities (Table S1), the total concentration of Al^3+^ in the NASICON-type phase was above 87%.

**Figure 5:**
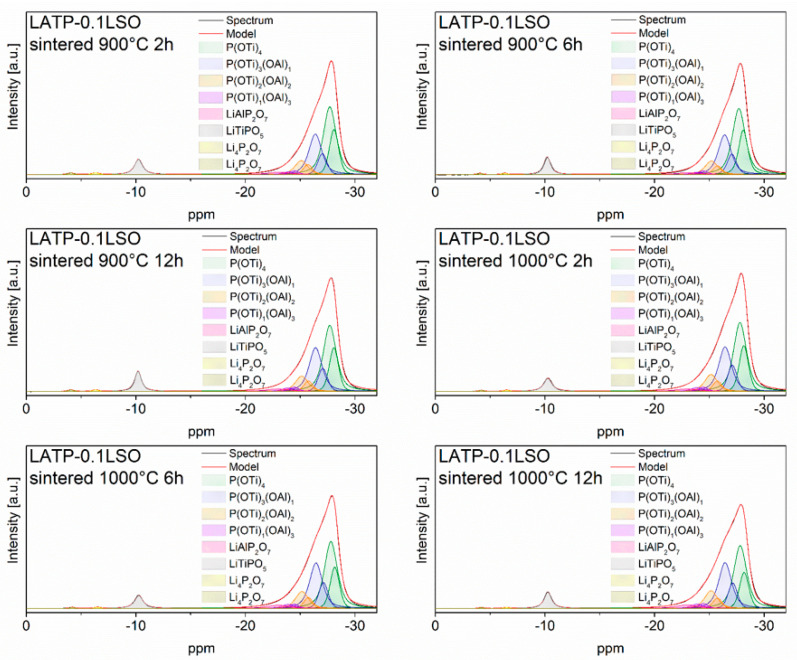
^31^P MAS NMR spectra of LATP–0.1LSO sintered at 900 and 1000 °C for 2, 6, or 12 h. The experimental and simulated spectra are displayed as black and red lines, respectively. The simulated spectra is the sum of distinct line-shapes displayed as colored lines.

^31^P MAS NMR spectra (Figure 5) of sintered LATP–0.1LSO ceramics mainly consist of a highly asymmetric peak located near −27 ppm. It may be deconvoluted into nine overlapping lines, located at: −28.1, −27.7, −27.1, −26.4, −25.7, −25.2, −24.5, −23.9, and −23.0 ppm. The signal at −23.0 ppm could be attributed to the LiAlP_2_O_7_ phase [24,42,43], as also detected in ^27^Al MAS NMR spectra. The previous resonances could be divided in two groups. The first one (LATP #1) contains lines at −28.1, −27.1, −25.7, and −24.5 ppm, while the other one (LATP #2) includes the signals located at −27.7, −26.4, −25.2, and −23.9. Both groups are assigned to a phosphorus environment P(OTi)_4−n_(OAl)_n_ (where n = 0, 1, 2, and 3) of the LATP phases with slightly different compositions [24,42]. Besides the discussed signals, three additional resonances at −10.2, −6.4, and −4.1 ppm were also detected. They can be assigned to the lithium-ion conductors, namely the LiTiPO_5_ and Li_4_P_2_O_7_ (triclinic) phases [24,41,42,43,49]. It is worth noting that the formation of the LiTiPO_5_ phase was also confirmed by XRD investigations.

Considering the spectra of materials sintered at different temperatures and times, all seem to be very similar. However, more closer analyses show changes in the integrated intensity of the P(OTi)_4−n_(OAl)_n_ coordinations. A comparison of signal integrated intensities is presented in Table S2. The signal at −28.1 ppm (LATP #1) or −27.7 ppm (LATP #2), assigned to the phosphorus P(OTi)_4_ environment, decreased, while the one ascribed to P(OTi)_3_(OAl)_1_ increased. Further changes were observed for the LATP #1 phase, where phosphorus P(OTi)_2_(OAl)_2_ and P(OTi)_1_(OAl)_3_ coordinations remained the same when a material was sintered at the same temperature but at different times. For the LATP #2 phase, there were no significant changes in the signals assigned to the P(OTi)_2_(OAl)_2_ and P(OTi)_1_(OAl)_3_ environments regardless of the technological process. Moreover, the relative integrated intensities of the signals attributed to the ^31^P bands for secondary phases were nearly the same. According to this analysis, we conclude that part of Al^3+^ may diffuse into LATP grains and substitute Ti^4+^ ions. To verify such a conclusion, we performed the calculations required to deduce the concentration of Al^3+^ ions in NASICON phases according to the following formula [5,6,24]:

(1) $$\frac{Al^{3+}}{Ti^{4+}}=\frac{4I_{4}+3I_{3}+2I_{2}+I_{1}}{4I_{0}+3I_{1}+2I_{2}+I_{3}}=\frac{x}{2-x}$$

where *I_n_* (n = 0, 1, 2, 3, and 4) represents the relative signal intensity of ^31^P bands associated with P(OTi)_4−n_(OAl)_n_ environments. The calculated values are presented in Table S2. One may notice that the calculated values of the Al^3+^ in LATP grains are slightly higher than the nominal one. For the highest sintering temperature, the concentration of aluminum ions further increased. This observation may be ascribed to the reaction of NASICON powder with the alumina crucible. During annealing, some of the Al^3+^ ions were incorporated into the material and finally diffused into grains.

**Figure 6:**
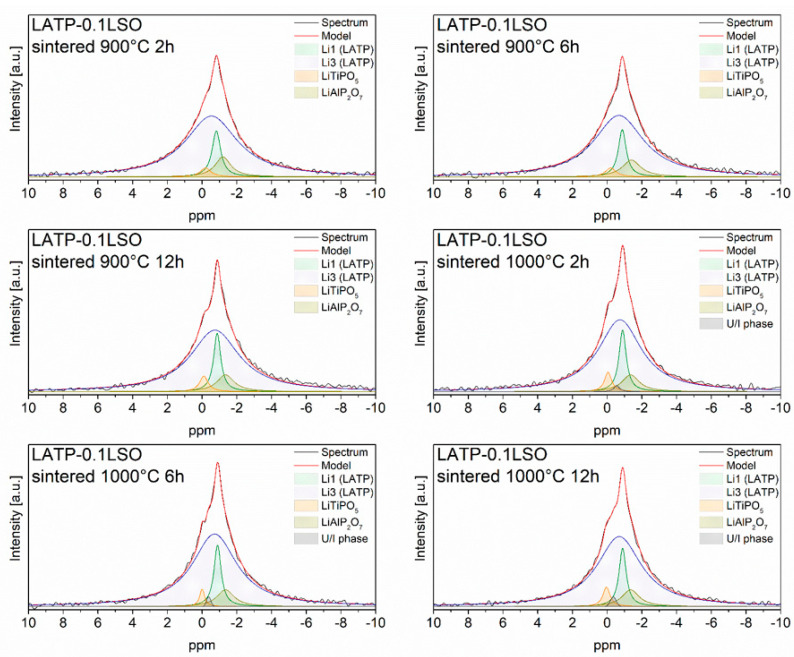
^6^Li NMR spectra of LATP–0.1LSO sintered at 900 and 1000 °C for 2, 6, or 12 h. The experimental and simulated spectra are displayed as black and red lines, respectively. The simulated spectra are the sum of distinct line-shapes displayed with different colors.

^6^Li (I = 1) MAS NMR spectra for LATP–0.1LSO ceramic material are presented in Figure 6. As a consequence of small dipolar and quadrupolar interactions, only central (CT) transitions of ^6^Li MAS NMR spectra were detected. Each spectrum consisted of the signals located at −1.4, −0.9, −0.7, −0.4, and −0.1 ppm. The resonances at −0.9 and −0.7 ppm may be assigned to Li1 and Li3 sites in the NASICON crystal structure [5,24,44]. The relative integrated intensity of the peak at −0.7 ppm was much higher than for the −0.9 ppm peak, in agreement with the preferential occupation of Li3 sites. The lines located at −1.4, −0.4, and −0.1 ppm can be attributed to the lithium environment in LiAlP_2_O_7_, LiTiPO_5_, and in the unidentified phase, respectively [24]. Referring to relative signal intensities, one may deduce that the concentration of lithium ions in the NASICON-type phase was about 88%. The relative integrated intensities of the signals remained the same regardless of the sintering temperature and time.

**Figure 7:**
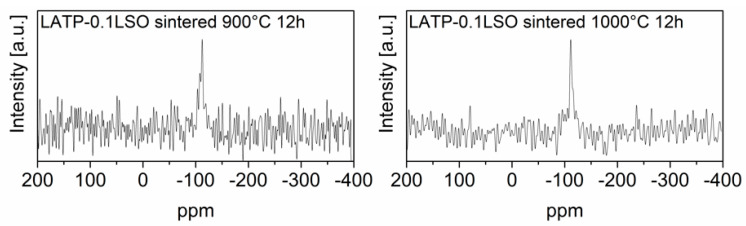
^29^Si MAS NMR spectra of LATP-0.1LSO sintered at 900 °C or 1000 °C for 12 h. The experimental spectra are displayed as black lines.

^29^Si MAS NMR spectra for LATP–0.1LSO sintered at 900 °C or 1000 °C are presented in Figure 7. They consisted mainly of a sharp peak around −112 ppm. It may be assigned to Si(OSi)_4_ units and ascribed to the SiO_2_ compound [50]. For the material sintered at 900 °C for 12 h, an additional weak resonance was detected around −108 ppm. Its presence may be associated with Si(OSi)_3_(OAl)_1_ units, where aluminum ions are incorporated to the material from the alumina crucible. Considering ^29^Si MAS NMR spectra and XRD results, we conclude that the Li_4_SiO_4_ additive material decomposed into Li_2_O and SiO_2_. In parallel, ^31^P MAS NMR spectra also showed the incorporation of additional Al^3+^ ions into NASICON grains, substituting Ti^4+^ ions. However, to perform such substitution, lithium ions should also diffuse into the grains of LATP. Thus, the Li_2_O product of the decomposition of Li_4_SiO_4_ may fulfill two prominent roles, namely diffusion into the LATP grains and participation in the formation of both LiTiPO_5_ and LiAlP_2_O_7_ compounds.

### 3.5. Impedance Spectroscopy

**Figure 8:**
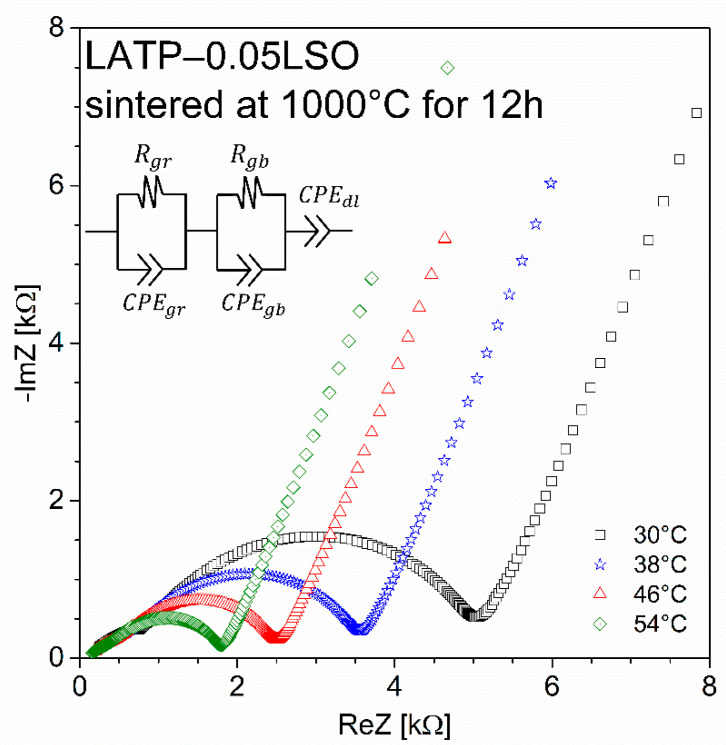
Nyquist plots for the data collected at 30 °C, 38 °C, 46 °C, and 54 °C for LATP–0.05LSO composite sintered at 1000 °C for 12 h. The equivalent circuit modeling the electrical properties of the sintered materials is displayed as inset.

The exemplary Nyquist plots collected at 30 °C for LATP-0.05LSO sintered at 1000 °C for 12 h is presented in Figure 8. It consists of two almost regular semicircles followed by a spur. From the shape of this plot, it may be concluded that the transport of lithium ions takes place through two different media, namely the grain interior and grain boundaries. The electrical properties can be analyzed in terms of the equivalent electrical circuit composed of two loops connected in series. Each loop includes a resistor R shunted by a constant phase element CPE and corresponds to the semicircles shown in the Nyquist plot. The high frequency loop represents the grain interior, while the second low frequency loop corresponds to the grain boundary contribution. 

The values of the apparent grain and total conductivity can be calculated by employing the equation *σ = L/(R·A)*, where *L* and *A* represent the sample thickness and electrode area. To evaluate these values, from the intersection of each semicircle with the Re Z axis, one may obtain the values of resistance: *R_gr_* and *R_tot_* = *R_gr_* + *R_gb_*. Due to unknown precise values of the size of the grains and the thickness of the grain boundary region, the values of the conductivities should be considered as apparent ones. The values of bulk (*σ_gr_*) and total (*σ_tot_*) ionic conductivities with their activation energies are presented in Table S4. The analysis of those results reveals that: (i) *σ_gr_* and *σ_tot_* increase with the additive concentration, and (ii) sintering temperature and time have a negligible influence on the values of *σ_gr_* and *σ_tot_* for the same concentration of LSO. According to (i), the enhancement of *σ_gr_* with higher additive content results from an increased lithium-ion concentration in LATP grains, proven by MAS NMR studies, while the *σ_tot_* increases due to the presence of secondary lithium-ion conducting phases (LiTiPO_5_ and LiAlP_2_O_7_). However, further modification of the sintering process did not lead to the enhancement of the total ionic conductivity, providing the maximum value of *σ_tot_* ca. 1.4 × 10^−4^ S∙cm^−1^. Referring to XRD and MAS NMR results, the Li_4_SiO_4_ additive material decomposed to the Li_2_O and SiO_2_ compounds. Therefore, further improvement of electrical properties may be hindered by the occurrence of SiO_2_ at grain boundaries, which impedes the transport of Li^+^ between the grains, as this material is not a lithium-ion conductor. 

**Figure 9:**
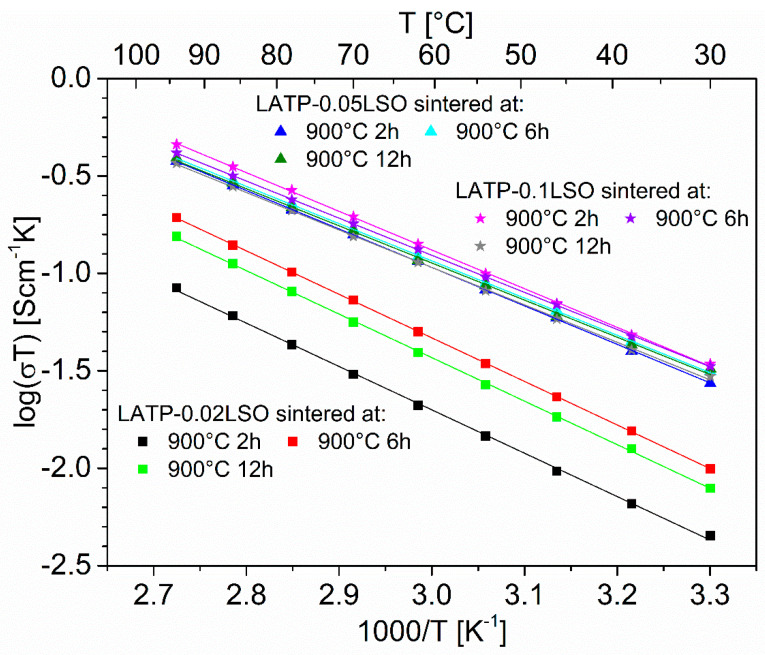
Arrhenius plots of the total ionic conductivity of the LATP–xLSO composites sintered at 900 °C for 2 h, 6 h, or 12 h.

The temperature-dependent total ionic conductivity results for LATP-LSO ceramics fulfills an Arrhenius dependence (Figure 9). The values of activation energy for the total (*E_tot_*) and grain (*E_gr_*) conductivity are listed in Table S4. They did not depend on sintering temperature and time. For ceramics sintered at 900 °C or 1000 °C, *E_gr_* varied from 0.29 to 0.32 eV, while for the material sintered at lower temperatures, its value was higher at ca. 0.34 eV. Considering *E_tot_*, one may notice that this value was nearly the same for LATP-LSO sintered at 800 °C and it decreased to ca. 0.39 eV when the composite was sintered at 900 °C or 1000 °C. This may be related to the slightly different composition of the material when sintered at 800, 900, or 1000 °C. Based on this fact, different values were reported for grain and total activation energy [5,7,9,12,14,17,18,19,23,24,26,28,29,30,35,48].

## 4. Discussion

The analysis of the obtained results reveal that the addition of LSO to the LATP with subsequent sintering at high temperatures results in highly conductive ceramics. Referring to our previous results concerning LATP-based ceramics without any additive material [23,24], we observed the increase in the total ionic conductivity value at about one order of magnitude. To understand such improvement of the electrical properties of the obtained ceramic composites, we decided to examine the samples employing SEM, HTXRD, and MAS NMR methods. Firstly, the increase in the total ionic conductivity was partially due to favorable changes in the microstructure while sintering. As revealed by SEM images, the average grain size increased with the sintering temperature, grains became more densely packed, and, as a result, the porosity was considerably reduced. 

Secondly, as evidenced by HTXRD and MAS NMR data, the enhancement of the total ionic conductivity may be related to changes in the secondary phase composition at grain boundaries during heat treatment. Based on HTXRD results, at ca. 800 °C, we observed the formation of the LiTiPO_5_ compound, which was not present in the base LATP material. ^31^P MAS NMR results also confirmed the presence of this compound in the composite material. Besides the formation of the LiTiPO_5_ phase, MAS NMR data reveal the presence of additional resonances ascribed to Li_4_P_2_O_7_ and LiAlP_2_O_7_ compounds, and also to another unidentified phase. The signal attributed to the LiAlP_2_O_7_ phase was also noticeable in ^27^Al MAS NMR spectra.

Moreover, after the thorough inspection of ^27^Al and ^31^P MAS NMR spectra, two slightly different compositions of NASICON-type phases were found. The detection of two ^27^Al and ^31^P MAS NMR patterns indicates that the Al^3+^ distribution in the LATP material is not homogenous. We assume that this observation may be attributed to the technological process to which the materials were subjected to. The mixture of reagents for synthesis and as-formed pellets for sintering were placed in an alumina crucible to complete the synthesis process. In such a case, the NASICON powder may have reacted with the alumina crucible at high temperatures. As a result, part of the aluminum ions may have diffused into the LATP grains and substituted for titanium ions. Further examination of such replacement revealed that the calculated concentration of Al^3+^ in LATP grains was slightly higher than the nominal one. Based on the above-described results and the presence of two slightly different Li_1+x_Al_x_Ti_2−x_(PO_4_)_3_ phases, the formation of a core-shell grain structure with an external surface enriched by Al^3+^ ions is probable and may account for the enhancement of the electrical properties of LATP-based ceramics.

It must be noted that to enable the Al^3+^ → Ti^4+^ substitution, some part of the lithium ions should also diffuse into the LATP grains to satisfy the conservation of the charge. In our case, the Li_4_SiO_4_ additive probably acted as a source of Li^+^ ions. As evidenced by HTXRD and ^29^Si MAS NMR investigations, the Li_4_SiO_4_ compound decomposed into Li_2_O and SiO_2_ at high temperatures. We assume that Li_2_O also participated in the formation of LiTiPO_5_ and LiAlP_2_O_7_ secondary phases. Hence, the employment of Li_4_SiO_4_ as a sintering aid should affect not only the grain boundary conductivity but also the grain (bulk) conductivity due to an increased concentration of lithium ions in the LATP grains. Conversely, the presence of SiO_2_ as a result of the LSO decomposition impedes any further increase of the total ionic conductivity in the studied materials. Therefore, the reported study clearly shows that it is crucial to eliminate or reduce the concentration of all non-conducting phases in the composite material in order to further improve its electrical properties.

## 5. Conclusions

Modifying the properties of the grain-boundary region with appropriate sintering aids is one of the possible approaches to obtain highly conductive ceramics. In this study, we prepared the Li_1.3_Al_0.3_Ti_1.7_(PO_4_)_3_−*x*Li_4_SiO_4_ (0.02 ≤ *x* ≤ 0.1) composite ceramic with the total ionic conductivity values exceeding 10^−4^ S∙cm^−1^, which is higher than for the pristine ceramic LATP material. Based on experimental evidence, we discerned two dominant factors affecting the total ionic conductivity of LATP-LSO ceramics. The first concerns the material’s microstructure, which changed as a function of sintering process parameters, especially temperature (the size of the grains increased with sintering temperature and simultaneously the thickness of the grain boundary layer decreased). The second is related to the formation of other lithium-ion conductors at the grain boundaries in the presence of LSO. HTXRD and MAS NMR studies revealed the formation of LiTiPO_5_, LiAlP_2_O_7_, and also another unidentified phase. Besides them, according to XRD and ^29^Si MAS NMR investigations, after sintering, there is a SiO_2_ phase formed due to the decomposition of the Li_4_SiO_4_ additive material. The presence of silicon dioxide at the grain boundaries impedes the Li^+^ conduction. Therefore, to ensure the further increase of total ionic conductivity in NASICON-based materials, it is essential to eliminate the non-conducting phases while keeping the lithium-ion conducting phases.

## Data Availability

Data sharing not applicable.

## Supplementary Materials

The following are available online at https://www.mdpi.com/article/10.3390/ma14195729/s1, Figure S1: SEM images taken in SE mode of the LATP–0.02LSO sintered at 800 °C for 2 h (a) and 1000 °C for 12 h (b), and LATP–0.1LSO sintered at 800 °C for 2 h (c) and 1000 °C for 12 h (d); Table S1: Relative integrated intensities (II) in percentage, full widths at half maximum height (FWHM) in ppm, and isotropic chemical shifts (δ) of the line-shapes used to simulate ^27^Al MAS NMR spectra, as shown in Figure 4 for the LATP–0.1LSO sample sintered under different conditions; Table S2: Relative integrated intensities (II) in percentage, full widths at half maximum height (FWHM) in ppm, and isotropic chemical shifts (δ) of the line-shapes used to simulate ^31^P MAS NMR spectra, as shown in Figure 5 for the LATP–0.1LSO sample sintered under different conditions. Actual (x_NMR_) Al^3+^ concentration in the LATP phase is also given along with the nominal one (x_NOM_); Table S3: Relative integrated intensities (II) in percentage, full widths at half maximum height (FWHM) in ppm, and isotropic chemical shifts (δ) of the line-shapes used to simulate the ^6^Li MAS NMR spectra shown in Figure 6 for the LATP–0.1LSO sample sintered under different conditions; and Table S4: Values of bulk (*σ_gr_*) and total (*σ_tot_*) ionic conductivities at 30 °C, as well as the bulk (*E*_gr_) and total (*E*_tot_) activation energies.
