# Supplementary material for: Electrical and Structural Properties of Li1.3Al0.3Ti1.7(PO4)3—Based Ceramics Prepared with the Addition of Li4SiO4
Source: Materials (Basel). 2021 Sep 30;14(19):5729. doi: 10.3390/ma14195729 (PMC8510155; doi:10.3390/ma14195729)
Supplement: Supplementary file 1 [file materials-14-05729-s001.zip › materials-1373237-supplementary.pdf]

materials

MDPI

Supplementary

Supplementary Material for Electrical and Structural Properties of $Li_{1.3}Al_{0.3}Ti_{1.7}(PO_4)_3$-Based Ceramics Prepared with the Addition of Li_4SiO_4

Konrad Kwatek [1,*], Wioleta Ślubowska [1], Jan Nowiński [1], Agnieszka Krawczyńska [2], Isabel Sobrados [3] and Jesús Sanz [3]

[1] Faculty of Physics, Warsaw University of Technology, 00-662 Warsaw, Poland; wioleta.slubowska@pw.edu.pl (W.Ś.); jan.nowinski@pw.edu.pl (J.L.N.)
[2] Faculty of Materials Science and Engineering, Warsaw University of Technology, 02-507 Warsaw, Poland; agnieszka.krawczynska@pw.edu.pl
[3] National Research Council, The Materials Science Institute of Madrid (ICMM CSIC), 28049 Madrid, Spain; isobrado@icmm.csic.es (I.S.); jsanz@icmm.csic.es (J.S.)
* Correspondence: konrad.kwatek@pw.edu.pl

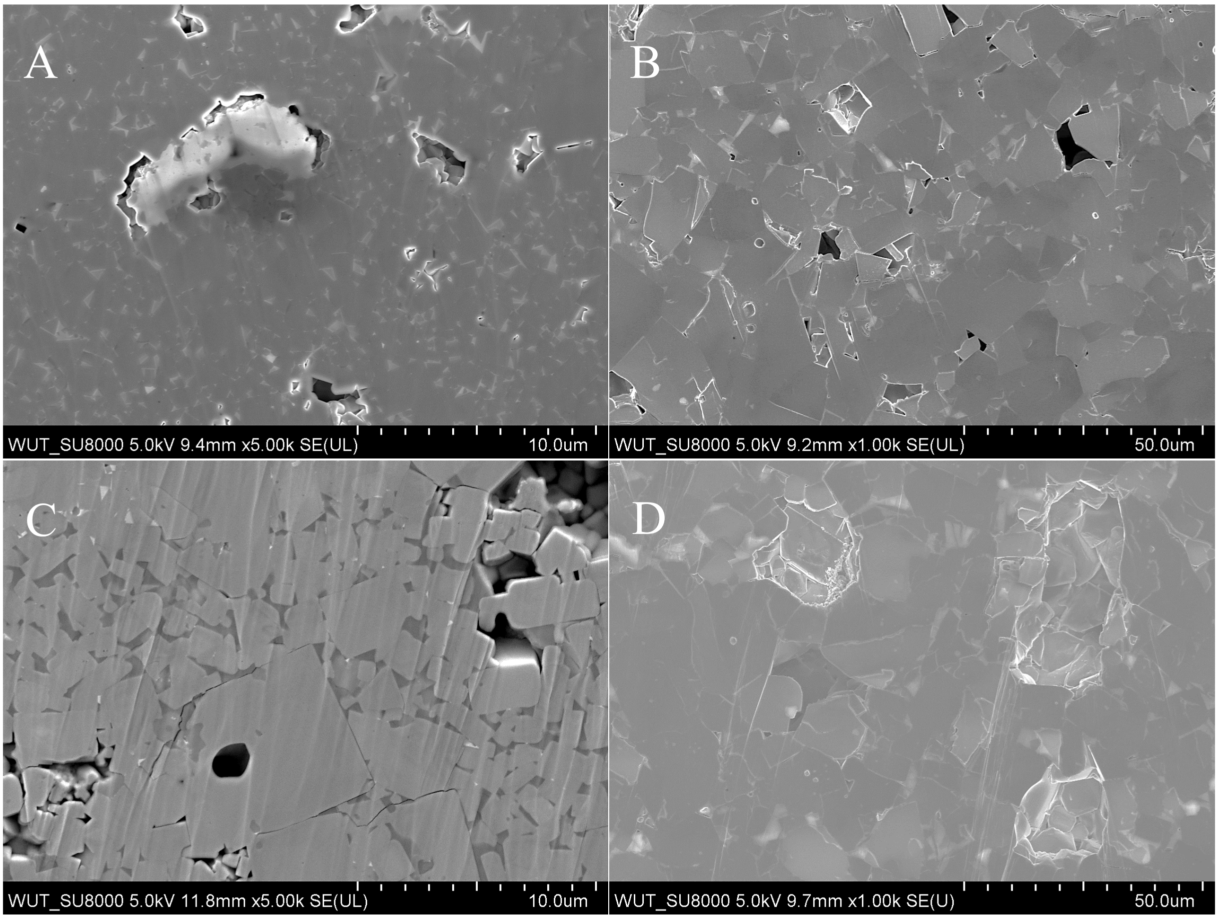

Figure S1. SEM images taken in SE mode of the LATP–0.02LSO sintered at 800 °C for 2 h (**A**) and 1000 °C for 12 h (**B**), and LATP–0.1LSO sintered at 800 °C for 2 h (**C**) and 1000°C for 12 h (**D**).

Table S1. Relative integrated intensities (II) in %, full widths at half maximum height (FWHM) in ppm and isotropic chemical shifts (δ) of the lineshapes used to simulate ^{27}Al MAS NMR spectra shown in Figure 4 for LATP–0.1LSO sample sintered under different conditions.

	AlO_6 (LATP1)			AlO_6 (LATP2)			AlO_6 ($LiAlP_2O_7$)			AlO_6 (U/I Phase)		
Sintering Conditions	δ [ppm]	FWHM [ppm]	II [%]	δ [ppm]	FWHM [ppm]	II [%]	δ [ppm]	FWHM [ppm]	II [%]	δ [ppm]	FWHM [ppm]	II [%]
900 °C 2 h	−14.60	3.18	46.9	−16.30	3.18	40.6	−19.60	6.40	12.5	–	–	–
900 °C 6 h	−14.60	3.16	46.3	−16.30	3.16	41.7	−19.60	6.40	12.0	–	–	–
900 °C 12 h	−14.50	3.16	49.6	−16.24	3.16	45.3	−19.60	6.40	5.1	–	–	–
1000 °C 2 h	−14.70	3.16	46.8	−16.40	3.16	43.6	−19.60	6.40	9.6	–	–	–
1000 °C 6 h	−14.60	3.16	48.3	−16.40	3.16	44.0	−19.60	6.40	7.7	–	–	–
1000 °C 12 h	−14.70	3.12	44.9	−16.40	3.12	42.7	−19.60	6.40	10.7	−18.90	2.00	1.8

Table S2. Relative integrated intensities (II) in %, full widths at half maximum height (FWHM) in ppm and isotropic chemical shifts (δ) of the lineshapes used to simulate ^{31}P MAS NMR spectra shown in Figure 5 for LATP–0.1LSO sample sintered under different conditions. Actual (xNMR) Al^{3+} concentration in LATP phase is also given along with the nominal one (xNOM).

	Composition		$P(OTi)_4$ (LATP1)			$P(OTi)_3(OAl)_1$			$P(OTi)_2(OAl)_2$			$P(OTi)_1(OAl)_3$		
sintering conditions	x_{NOM}	x_{NMR}	δ [ppm]	FWHM [ppm]	II [%]	δ [ppm]	FWHM [ppm]	II [%]	δ [ppm]	FWHM [ppm]	II [%]	δ [ppm]	FWHM [ppm]	II [%]
900 °C 2 h	0.30	0.32	−27.70	1.40	34.7	−26.38	1.40	20.8	−25.18	1.40	7.3	−23.90	1.40	1.5
900 °C 6 h	0.30	0.31	−27.70	1.40	34.4	−26.40	1.40	20.7	−25.18	1.40	7.2	−23.95	1.40	1.3
900 °C 12 h	0.30	0.34	−27.70	1.40	32.9	−26.40	1.40	21.8	−25.14	1.40	7.7	−23.95	1.40	1.8
1000 °C 2 h	0.30	0.34	−27.80	1.40	33.3	−26.44	1.40	21.5	−25.16	1.40	8.2	−23.95	1.40	2.0
1000 °C 6 h	0.30	0.36	−27.80	1.40	32.7	−26.44	1.40	22.2	−25.16	1.40	8.3	−23.90	1.40	2.1
1000 °C 12 h	0.30	0.38	−27.82	1.40	31.5	−26.44	1.40	22.9	−25.16	1.40	8.9	−23.90	1.40	2.2

	Composition		$P(OTi)_4$ (LATP2)			$P(OTi)_3(OAl)_1$			$P(OTi)_2(OAl)_2$			$P(OTi)_1(OAl)_3$		
sintering conditions	x_{NOM}	x_{NMR}	δ [ppm]	FWHM [ppm]	II [%]	δ [ppm]	FWHM [ppm]	II [%]	δ [ppm]	FWHM [ppm]	II [%]	δ [ppm]	FWHM [ppm]	II [%]
900 °C 2 h	0.30	0.32	−28.08	1.00	14.7	−27.02	1.00	6.9	−25.70	1.00	3.3	−24.45	1.00	1.5
900 °C 6 h	0.30	0.31	−28.10	1.00	16.0	−27.06	1.00	7.3	−25.75	1.00	3.2	−24.45	1.00	1.5
900 °C 12 h	0.30	0.34	−28.07	1.00	13.9	−27.06	1.00	7.3	−25.75	1.00	3.4	−24.45	1.00	1.6
1000 °C 2 h	0.30	0.34	−28.15	1.00	14.1	−27.10	1.00	8.1	−25.74	1.00	3.2	−24.45	1.00	1.5
1000 °C 6 h	0.30	0.36	−28.15	1.00	12.9	−27.12	1.00	8.1	−25.74	1.00	3.4	−24.45	1.00	1.6
1000 °C 12 h	0.30	0.38	−28.18	1.00	11.5	−27.14	1.00	8.2	−25.74	1.00	3.4	−24.45	1.00	1.8

	$LiAlP_2O_7$			$LiTiPO_5$			$Li_4P_2O_7$ (Triclinic)			$Li_4P_2O_7$ (Triclinic)		
sintering conditions	δ [ppm]	FWHM [ppm]	II [%]	δ [ppm]	FWHM [ppm]	II [%]	δ [ppm]	FWHM [ppm]	II [%]	δ [ppm]	FWHM [ppm]	II [%]
900 °C 2 h	−23.05	2.80	2.7	−10.25	1.01	5.6	−6.35	0.70	0.6	−4.05	0.70	0.6
900 °C 6 h	−23.10	2.80	2.3	−10.20	0.75	5.5	−6.35	0.60	0.4	−4.05	0.60	0.4
900 °C 12 h	−23.00	2.80	2.8	−10.20	0.75	6.0	−6.35	0.67	0.4	−4.05	0.67	0.4
1000 °C 2 h	−23.10	2.80	2.8	−10.20	0.92	4.7	−6.35	0.60	0.3	−4.05	0.60	0.3
1000 °C 6 h	−23.00	2.80	3.5	−10.27	0.92	4.8	−6.50	0.60	0.3	−4.20	0.60	0.3
1000 °C 12 h	−23.00	2.80	3.8	−10.27	0.80	5.3	−6.50	0.60	0.2	−4.20	0.60	0.2

Table S3. Relative integrated intensities (II) in %, full widths at half maximum height (FWHM) in ppm and isotropic chemical shifts (δ) of the lineshapes used to simulate the ^{6}Li MAS NMR spectra shown in Figure 6 for LATP–0.1LSO sample sintered under different conditions.

	Li1 (LATP)			Li3 (LATP)			$LiTiPO_5$			$LiAlP_2O_7$			U/I Phase		
sintering conditions	δ [ppm]	FWHM [ppm]	II [%]	δ [ppm]	FWHM [ppm]	II [%]	δ [ppm]	FWHM [ppm]	II [%]	δ [ppm]	FWHM [ppm]	II [%]	δ [ppm]	FWHM [ppm]	II [%]
900 °C 2 h	−0.82	0.60	10.8	−0.54	3.65	78.2	−0.20	0.75	2.6	−1.20	1.10	8.5	–	–	–
900 °C 6 h	−0.87	0.55	10.2	−0.68	3.50	78.7	−0.20	0.75	2.7	−1.40	1.30	8.5	–	–	–
900 °C 12 h	−0.87	0.50	11.5	−0.74	3.40	76.9	−0.1	0.60	3.7	−1.35	1.20	7.9	–	–	–
1000 °C 2 h	−0.89	0.50	11.6	−0.74	3.00	76.3	−0.05	0.50	3.7	−1.35	1.20	7.5	−0.55	0.40	0.9
1000 °C 6 h	−0.89	0.50	11.4	−0.72	3.10	78.2	0.00	0.35	2.3	−1.35	1.20	7.4	−0.38	0.30	0.7
1000 °C 12 h	−0.89	0.50	11.0	−0.70	3.10	76.8	0.04	0.50	3.7	−1.35	1.20	7.5	−0.35	0.40	1.0

Table S4. Values of bulk (σ_{gr}) and total (σ_{tot}) ionic conductivities at 30°C as well as the bulk (E_{gr}) and total (E_{tot}) activation energies.

Composition	Sintering Conditions	σ_{gr} (30°C) [S·cm^{-1}]	σ_{tot} (30°C) [S·cm^{-1}]	E_{gr} [eV]	E_{tot} [eV]
LATP-0.02LSO	800 °C 2 h	1.4×10^{-4}	1.3×10^{-5}	0.34	0.44
	800 °C 6 h	3.6×10^{-4}	4.2×10^{-5}	0.34	0.44
	800 °C 12 h	2.8×10^{-4}	2.6×10^{-5}	0.34	0.44
	900 °C 2 h	1.8×10^{-4}	1.5×10^{-5}	0.34	0.44
	900 °C 6 h	3.1×10^{-4}	3.3×10^{-5}	0.35	0.44
	900 °C 12 h	2.6×10^{-4}	2.6×10^{-5}	0.35	0.44
	1000 °C 2 h	2.7×10^{-4}	2.1×10^{-5}	0.35	0.44
	1000 °C 6 h	2.1×10^{-4}	1.7×10^{-5}	0.34	0.43
	1000 °C 12 h	2.5×10^{-4}	3.5×10^{-5}	0.35	0.43

Composition	Sintering Conditions	σ_{gr} (30°C) [S·cm^{-1}]	σ_{tot} (30°C) [S·cm^{-1}]	E_{gr} [eV]	E_{tot} [eV]
LATP-0.05LSO	800 °C 2 h	6.9×10^{-4}	1.2×10^{-4}	0.31	0.40
	800 °C 6 h	5.2×10^{-4}	8.6×10^{-5}	0.32	0.40
	800 °C 12 h	4.4×10^{-4}	5.3×10^{-5}	0.31	0.40
	900 °C 2 h	5.3×10^{-4}	9.0×10^{-5}	0.31	0.39
	900 °C 6 h	7.2×10^{-4}	1.1×10^{-4}	0.30	0.38
	900 °C 12 h	6.1×10^{-4}	1.1×10^{-4}	0.31	0.38
	1000 °C 2 h	4.9×10^{-4}	8.2×10^{-5}	0.29	0.38
	1000 °C 6 h	5.2×10^{-4}	8.5×10^{-5}	0.29	0.39
	1000 °C 12 h	5.0×10^{-4}	9.3×10^{-5}	0.30	0.39

Composition	Sintering Conditions	σ_{gr} (30°C) [S·cm^{-1}]	σ_{tot} (30°C) [S·cm^{-1}]	E_{gr} [eV]	E_{tot} [eV]
LATP-0.1LSO	800 °C 2 h	7.9×10^{-4}	1.4×10^{-4}	0.31	0.39
	800 °C 6 h	5.8×10^{-4}	1.2×10^{-4}	0.31	0.40
	800 °C 12 h	5.7×10^{-4}	1.1×10^{-4}	0.30	0.39
	900 °C 2 h	5.7×10^{-4}	1.1×10^{-4}	0.30	0.39
	900 °C 6 h	5.2×10^{-4}	1.1×10^{-4}	0.29	0.38
	900 °C 12 h	5.0×10^{-4}	9.8×10^{-5}	0.29	0.38
	1000 °C 2 h	5.5×10^{-4}	1.3×10^{-4}	0.29	0.38

	1000 °C 6 h	5.2×10^{-4}	1.4×10^{-4}	0.29	0.37
	1000 °C 12 h	4.8×10^{-4}	1.2×10^{-4}	0.29	0.38
